# Rheumatoid Arthritis: Applicability of Ready-to-Use Human Cartilaginous Cells for Screening of Compounds with TNF-Alpha Inhibitory Activity

**DOI:** 10.3390/biom10111563

**Published:** 2020-11-17

**Authors:** Larissa T. Volova, Evgeniy I. Pugachev, Victoria V. Rossinskaya, Violetta V. Boltovskaya, Dmitry A. Dolgushkin, Natalya Ossina

**Affiliations:** Institute of Experimental Medicine and Biotechnology, Samara State Medical University, Chapaevskaya St., 89, 443099 Samara, Russia; l.t.volova@samsmu.ru (L.T.V.); e.i.pugachev@samsmu.ru (E.I.P.); v.v.rossinskaya@samsmu.ru (V.V.R.); v.v.boltovskaya@samsmu.ru (V.V.B.); d.a.dolgushkin@samsmu.ru (D.A.D.)

**Keywords:** rheumatoid arthritis, cartilage, tumor necrosis factor assay, primary cell culture, interleukin-6

## Abstract

In the context of modern drug discovery, there is an obvious advantage to designing phenotypic bioassays based on human disease-relevant cells that express disease-relevant markers. The specific aim of the study was to develop a convenient and reliable method for screening compounds with Tumor Necrosis Factor-alpha (TNF-α) inhibitory activity. This assay was developed using cryopreserved ready-to-use cartilage-derived cells isolated from juvenile donors diagnosed with polydactyly. It has been demonstrated that all donor (10 donors) cells were able to respond to TNF-α treatment by increased secretion of pro-inflammatory cytokine IL-6 into subcultural medium. Inhibition of TNF-α using commercially available TNF-α inhibitor etanercept resulted in a dose-dependent decrease in IL-6 production which was measured by Enzyme-Linked Immunosorbent Assay (ELISA). TNF-α dependent IL-6 production was detected in the cells after both their prolonged cultivation in vitro (≥20 passages) and cryopreservation. This phenotypic bioassay based on ready-to-use primary human cells was developed for detection of novel TNF-α inhibitory compounds and profiling of biosimilar drugs.

## 1. Introduction

The use of TNF-α inhibitors in clinical practice is considered as one of the largest achievements of medicine in recent decades [1,2]. TNF-α causes inflammatory reactions in the body, which leads to such serious diseases as rheumatoid arthritis, ankylosing spondylitis, Crohn’s disease, psoriasis, refractory asthma and others. These diseases are successfully treated with TNF-α-inhibitors. The success of TNF-α-inhibitor drugs has sparked a boom in research for finding new molecules with TNF-α inhibitory activity [3,4]. Generally, the screening of new compounds is performed by in vitro assays based on using immortal cell lines. However, many immortal cell lines showed resistance to cytotoxicity, difference in membrane permeability, utilization of compounds, etc. Clinical failure of many promising compounds that were found by these assays led to the development of new-generation phenotypic bioassays based on human “normal” cells, expressing disease-relevant targets [5,6]. The physiology of “normal” cells has stronger similarity to cells in vivo than immortalized cells. One of the cell-based strategies for developing such phenotypic bioassays is using primary human cells isolated from disease-related tissue [5,7,8].

The first TNF-α evaluation bioassays were based on the measurement of cytotoxicity toward target cells of non-human origin such as murine L929 [9,10] or WEHI 164 [11,12]. Later, the uses of human cells for evaluation of TNF-α-mediated effects on cytotoxicity [13], NFkB activation [14], E-selectin expression [13,15], gene expression profile [16,17] were established.

Since the identified compounds may have diverse pharmacological effects depending on the cell type chosen for screening, it is important to use human cells in which a disease is manifested. In the animal model of arthritis, overexpression of TNF-α results in severe chronic inflammation of joints [18,19]. Inflammatory conditions of joints are accompanied by cartilage matrix destruction. Cartilage is an avascular tissue that consists of only two components—an extracellular matrix and cartilaginous cells. Cartilaginous cells are often presented in literature as uniform populations of chondrocytes. However, at least three phenotypic cells found in cartilage have been scientifically documented: chondroblasts or immature chondroblast-like cells, mature chondrocytes, and hypertrophic chondrocytes. They are different in their morphology, proliferative capacity, and their gene expression profile [20,21,22,23]. The first type—chondroblasts or chondroblast-like cells—are immature cells characterized by high proliferative potency. They are the source of the second type of cells—mature chondrocytes—which maintain the cartilage matrix. Differentiation of mature chondrocytes into postmitotic hypertrophic cells is followed by apoptosis of the third type of cell—hypertrophic chondrocytes—along with blood vessel invasion and the replacement of the cartilaginous matrix by bone tissue. Decades of work has been performed to investigate the signaling pathways that participate in physiological alterations of cartilage [21,24,25]. Based on these investigations, the disruption of the normal resting state of cartilaginous cells by TNF-α could be viewed as an injury response involving changes in expression of pro-inflammatory cytokines, matrix destructive proteases, and genes responsible for chondrophenotypic maturation and ossification. Therefore, the important role of cartilaginous cells in TNF-α-induced inflammation makes them an ideal model for the evaluation of compounds with TNF-α inhibitory activity. Choosing the right chondrocyte cell line for in vitro assays is a challenging topic that has been explored in recent studies: it has been reported that immortalized T/C-28a2 and SW-1353 chondrocyte cell lines are not always suitable as in vitro models for studies of classical inflammatory stimuli [26]. Authors concluded that primary human chondrocytes still remain the best effective in vitro cell system to study; for instance, the nitric oxide effect on cartilage. We showed that immature cartilaginous cells isolated from juvenile donors diagnosed with polydactyly could be used as TNF-α responding cells expressing disease-relevant inflammatory markers. Using expanded in vitro cryopreserved cartilaginous cells, we developed a convenient and reliable bioassay for both screening of novel TNF-α inhibitors and evaluation of biological activity of biosimilar drugs.

## 2. Materials and Methods

### 2.1. Materials

M199 medium (Biolot, Saint Petersburg, Russia); FCS (Biolot, Saint Petersburg, Russia); Gelatin (Biolot, Saint Petersburg, Russia); Gentamycin (Dalchempharm, Khabarovsk, Russia); Chemotrypsin (Biolot, Saint Petersburg, Russia); Versene solution (Biolot, Saint Petersburg, Russia); Collagenase (Biolot, Saint Petersburg, Russia); TNF-alpha (generous gift from Generium Pharmaceutical, Moscow, Russia); Etanercept (Pfizer Inc., New York, NY, USA); IL-6 ELISA (Vector Best, Novosibirsk, Russia); MTT (Sigma-Aldrich, Saint Louis, MO, USA); Cryovials (Nunc, Roskilde, Denmark). In addition, 96-well plates (TPP, Schaffhausen, Switzerland).

### 2.2. Isolation and Culturing of Immature Cartilaginous Cells

With approval of the Medical Ethics Committee of Samara State University (project identification code № 186, approval date: 10 April 2017) human immature cartilaginous cells were isolated from cartilage specimen of juvenile polydactyly-diagnosed donors undergoing supernumerary digit amputation surgery. The study was conducted in accordance with the Declaration of Helsinki and informed consent was obtained from the donor’s parents before participation in the study. The cartilage slices were harvested under sterile conditions from the phalange joints. The pieces were washed with sterile physiological saline and then incubated on a shaking platform at 37 °C for 30 min with serum-free M199 medium-0.2% collagenase. After incubation, the undigested cartilage pieces were washed and transferred to Petri dishes, where they were treated for 1 min with 160 ug/mL Chemotrypsin–0.02% Versene solution and then rapidly covered with M199 that contained 40% FCS. The pieces containing cartilaginous cells were then transferred to 25 cm^2^ flasks, coated with 0.1% gelatin. When the cartilaginous cells appeared out of the cartilage pieces, they were reseeded in the same flask for 1 more week (passage 0). The seeding of cartilaginous cells in monolayer was performed at density 3 × 10^4^ cells/cm^2^. The culture medium M199, supplemented with 10% FCS and gentamicin (88 ug/mL), was changed every 4–5 days. The cells were cultured up to 25–30 passages.

### 2.3. TNF-α Stimulation

Since the goal of this study was to develop a new cell-based assay, the experimental conditions of TNF-α stimulation included several protocol designs. In initial experiments, 100% confluent cartilaginous cells from a continuously growing culture were primed by serum deprivation for 24 h, then treated overnight with either 10 ng/mL TNF-α alone or in combination with commercially available TNF-α inhibitor Etanercept (Pfizer Inc., New York, NY, USA) 0.01 ng/mL to 0.1 ug/mL). The TNF-α with etanercept mixture was incubated in a serum-free medium for at least 30 min at room temperature before being added to the cells. The final assay protocol for TNF-α stimulation was as follows: cryopreserved, ready-to-use cells were seeded at density 2 × 10^4^ per well directly to the 96-well assay plates. On the third day, 2 h before TNF-α treatment, the medium was changed to the serum-free medium. Subsequently, each well—except those that served as controls—received 10 ng/mL TNF-α or a mixture of TNF-α (10 ng/mL) + etanercept (10 ng/mL). TNF-α and etanercept reagents were incubated together for 30 min at room temperature before being added to the cells. After overnight incubation, the cell medium was collected for assessment of IL-6 by ELISA. All assays were replicated (2 to 6 replicates) for each treatment: untreated control cells, TNF-α treated cells, and TNF-α + etanercept treated cells. This protocol was recommended for the quality control test of ready-to-use cells.

### 2.4. Enzyme-Linked Immunosorbent Assay (ELISA)

IL-6 protein level in conditioned medium was quantified using immunosorbent assay according to the manufacturer’s instructions (Vector, RUS). Briefly, 100 μL of the sample was added to a 96-well plate, which was coated with IL-6 antibody. Then, 100 μL of a biotin-labeled detection antibody was added to each well for 1 h at 37 °C. After washing with wash buffer, streptavidin-horseradish peroxidase conjugate was added to each well at 37 °C for 30 min and substrate reagent was added to each well for 15–25 min. The reaction was suspended by adding a stop solution to each well. Optical density was measured at 450 nm using spectrophotometer Thermo Multiscan FC (Thermo Scientific, Waltham, MA, USA). The IL-6 concentration (pg/mL) of the samples was calculated from the standard curve.

### 2.5. MTT Viability Test

Viability was evaluated using the 3-(4,5-dimethylthiazol-2-yl)-2,5-diphenyltetrazolium bromide (MTT) assay, which measures the ability of viable cells to convert a soluble tetrazolium salt to an insoluble purple formazan precipitate. Before TNF-α treatment, the cells were incubated without serum for 24 h, and then induced by TNF-α alone or by mixture of TNF-α and Enbrel^®^. After removing the medium, each well was incubated with 0.5 mg/mL MTT in a serum-free medium at 37 °C for 3.5 h. At the end of the incubation period, the medium was removed, and the intracellular formazan was solubilized with 200 μL DMSO and quantified by reading the absorbance at 550 nm on a microplate reader Tecan Infinite M200 Pro (Tecan, Mennedorf, Switzerland). The percentage of cell viability was calculated based on the absorbance measured relative to the absorbance of the untreated cells grown in medium containing 10% serum.

### 2.6. Cryopreservation

Expanded cells were harvested and resuspended in a cryopreservation medium optimized for the primary cartilaginous cell type: M199 media, containing 5% DMSO and 70% FCS. The cells were dispensed in cryovials (Nunc, Roskilde, Denmark) at density 1.5 million cells/mL. Assay ready cells were stored long term (months) in a liquid nitrogen freezer.

### 2.7. Thawing of Cryopreserved Ready-to-Use Cells

Frozen cells were thawed quickly in a water bath. Immediately after thawing, the cells were gently resuspended in medium M199, containing 10% serum, and seeded into 96-well assay plates. The next day, the cell medium was changed with a fresh medium and an assay was performed as described above.

### 2.8. Statistical Analysis

SPSS software (v.25, IBM, Armonk, NY, USA) was used for statistical analysis. Data were expressed as mean ± SD. Comparisons between groups were made by one-way ANOVA and two-way ANOVA. When significance was indicated, a Dunnett T3 post-hoc analysis was used. *p* value < 0.05 was considered statistically significant.

## 3. Results

### 3.1. Cartilage Tissue Specimen Obtained from Amputated Supernumerary Digits of Juvenile Donors Is an Attractive Source of Cartilaginous Cells for In Vitro Assays

**Figure 1:**
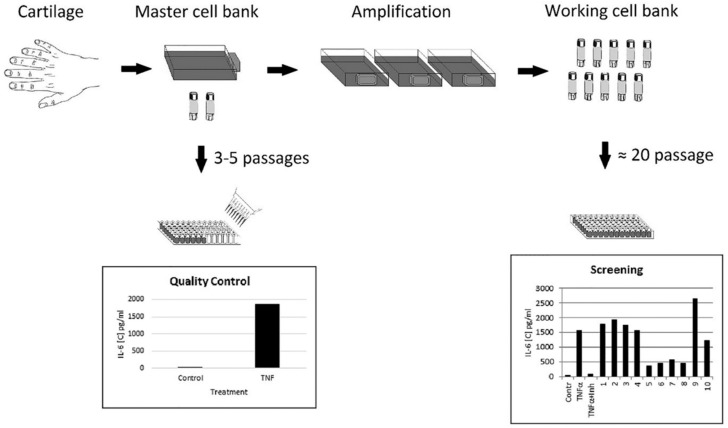
Design of the ready-to-use cell bioassay. Master and working cell banks could be obtained in advance of screening. Immature cartilaginous one-donor cells, which have been validated for quality (master cell bank), can be expanded and stored in liquid nitrogen (working cell bank) and then used routinely over extended periods of time for bioassays.

Cell-based assays require a large number of cells. Human primary cells isolated from the cartilage tissue of juvenile donors diagnosed with polydactyly is a reliable source of immature cartilaginous cells for in vitro studies due to their chondroblast-like potential to proliferate in vitro for a long time. Notably, 80% of confluent cultured cells had an average density of 1.5 × 10^4^ cells/cm^2^. From the cartilage specimen obtained from one donor, we calculated that, theoretically, 2.62 × 10^12^ cells can be obtained at passage 20. The possibility of providing a stable supply of amplified cells from a single donor is very attractive for ensuring the same quality of cells for screening bioassay. The goal of the study was to design a primary cartilaginous cell-based assay that would be compatible to generally applicable assays based on using immortal cell lines. Our approach in designing the assay is summarized in Figure 1.

First, cartilaginous cells of early passage level (Passages 3–5), validated by Quality Control test for their response to TNF-α stimulation, would be frozen to generate a master cell bank. Cells from the master bank could be expanded further (Passages 10–30) to create a working cell bank of ready-to-use, cryopreserved cells. Thus, our approach eliminated the need to continuously maintain cells in culture and allowed us to expand the cells to the quantity required for screening of a particular library of compounds. These ready-to-use cells could be easily moved to different laboratories interested in screening of TNF-α inhibitors.

### 3.2. Response of Immature Cartilaginous Cells to TNF-α Stimulation

**Figure 2:**
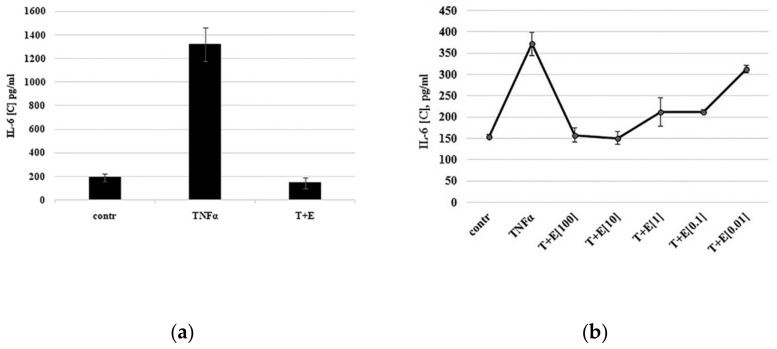
TNF-α-mediated expression of IL-6 by cartilaginous cells. (**a**) Cells were primed with serum deprivation for 24 h (contr.) and then treated with TNF-α alone (TNFα) or together with etanercept (T+E). During drug discovery screening, etanercept could be replaced by novel compounds. (**b**) Dose-dependent inhibition of IL-6 expression by TNF-α inhibitor etanercept. Cells were treated with 10 ng/mL TNF-α (TNFα) alone or together with etanercept (T+E) at concentrations from 0.01 to 100 ng/mL.

Using the immature cartilaginous cells described above, the expression of pro-inflammatory IL-6 cytokine in response to TNF-α treatment was evaluated by ELISA. As shown in Figure 2a, IL-6 expression was minimal in untreated cells (control) and its expression significantly increased in the presence of TNF-α (TNF-α).

To confirm that the observed changes in cytokine expression were due to TNF-α activity, commercially available TNF-α inhibitor etanercept was added to TNF-α to neutralize it. Indeed, TNF-α-mediated IL-6 expression was inhibited in the presence of etanercept to the control level (T+E). Thus, immature cartilaginous cells isolated from juvenile donors were able to express pro-inflammatory cytokine IL-6 in a TNF-α-dependent manner.

### 3.3. TNF-α Inhibitor Etanercept Inhibits IL-6 Expression in a Dose-Dependent Manner

Etanercept, a human dimeric fusion protein, functions as a TNF-α inhibitor by competitively binding to TNF-α and preventing its activation of the inflammatory cascade. To investigate whether TNF-α inhibitor etanercept inhibits IL-6 expression in a dose-dependent manner, the TNF-α inhibition assay was performed using different etanercept concentrations ranging from 0.01 to 100 ng/mL. As shown in Figure 2b, etanercept at a concentration of 0.01 ng/mL did not inhibit IL-6 expression, while starting from a concentration of 0.1 ng/mL, the expression of IL-6 was inhibited in a dose-dependent manner.

### 3.4. Evaluation of TNF-α-Mediated Effects on the Cells Obtained from 10 Donors

To confirm that immature cartilaginous cells isolated from different donors are able to respond to TNF-α in the same manner, TNF-α stimulation/inhibition was performed on cells obtained from 10 donors (Table 1).

**Figure 3:**
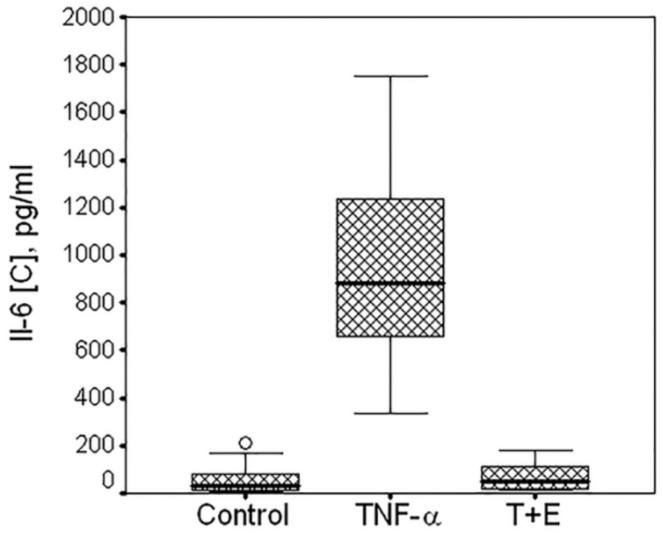
Box plot of the TNF-α-mediated expression of IL-6 by cartilaginous cells isolated from 10 donors. Data are from multiple experiments. Horizontal line in the box plot indicates median, low and upper borders of the box are quartiles. IL-6 production was measured in the medium of untreated cells (control), or cells treated with TNF-α (TNF-α) or with mixture of TNF-α and etanercept (T+E). Multiple comparisons (Dunnett T3 test) showed highly significant differences between control and TNF (*p* < 0.001); between TNF an TNF + E (*p* < 0.001); no difference between control and TNF + E (*p* = 0.994).

To examine interactions between groups (control, TNF-α, or TNF-α + E) and donors, two-way ANOVA was used. Effect of group: F = 948.0, *p* < 0.001; effect of donor: F = 13.7, *p* < 0.001. The data are summarized in Figure 3.

Without exception, the cells isolated from different polydactyl donors were showing highly significant increase in IL-6 expression in the presence of TNF-α (*p* < 0.001) and its inhibition in the presence of TNF-α inhibitor etanercept (*p* < 0.001). It should be noted that the box plot shown in Figure 3 was established on the data obtained from experiments with different experimental conditions such as different cell density, time of serum deprivation, reagents concentration, plate format, etc. Therefore, the high IL-6 expression variation was not only due to donor-to-donor variability but also reflects different experimental conditions.

### 3.5. Optimization of Assay

**Figure 4:**
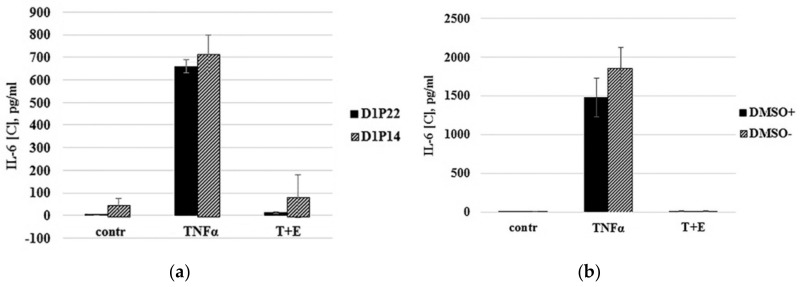

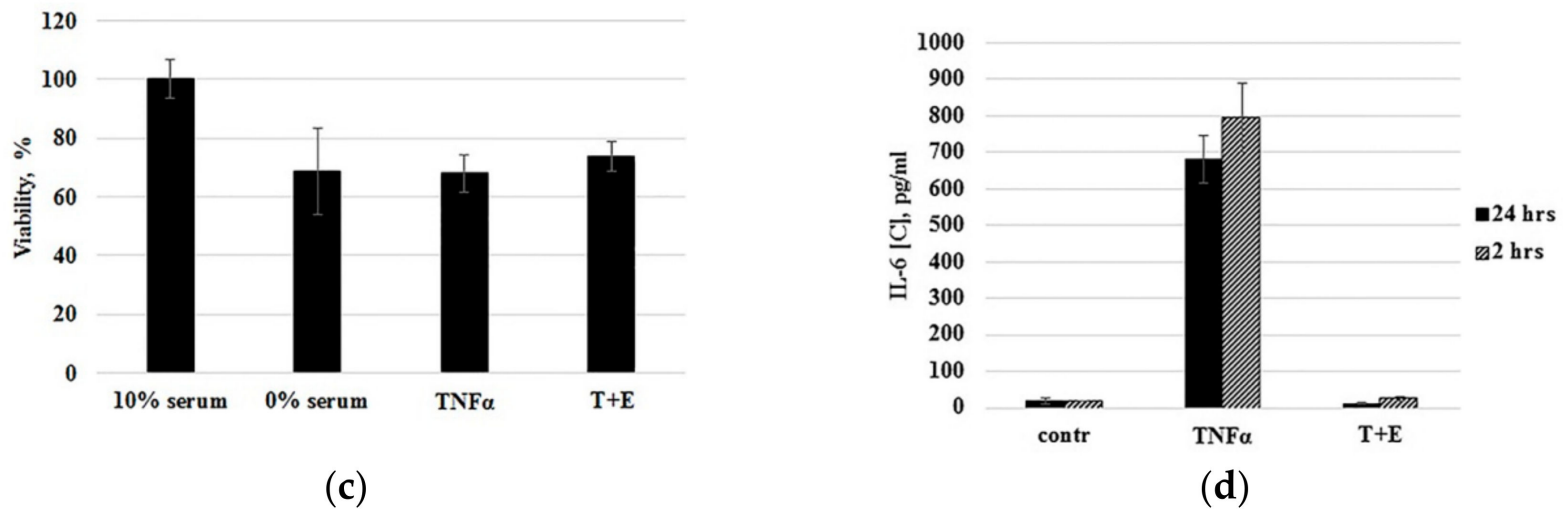
Optimization of ready-to-use cell bioassay. (**a**) Comparison of IL-6 expression by continuously subcultured in vitro (Passage 22) and cryopreserved (Passage 14) cartilaginous cells from the same donor. (**b**) TNF-α induced the response of ready-to-use cryopreserved cells due to different thawing conditions. Thawed cells were directly seeded to the wells (DMSO+) or washed out of DMSO by centrifugation before seeding (DMSO-). (**c**) MTT viability test. Cells were incubated in media with serum (10% serum) and no serum (0% serum) for 24 h. Serum-deprived cells were treated with TNF-α alone (TNFα) or with mixture of TNF-α and etanercept (T+E). (**d**) Comparison of IL-6 expression by cartilaginous cells primed with serum deprivation for 24 h and 2 h.

Primary cells often lose their characteristics over a long duration in vitro and are usually more susceptible to damage during the frozen storage. For example, the cultivation of chondrocytes as a monolayer culture results in rapid cell dedifferentiation that is accompanied with gene expression changes [27,28]. To investigate how both long-term cultivations in vitro and freezing of immature cartilaginous cells will affect their ability to respond to TNF-α treatment, TNF-α stimulation was performed using cells from the same donor after being subcultured for a long time to reach passages 14 and 22 (P14 and P22). While subculturing the cells to passage 22, the same cells of passage 14 were cryopreserved. As shown in Figure 4a, the immature cartilaginous cells of late passages (P14, P22)—before or after cryopreservation—were able to respond to TNF-α stimulation in the same manner.

Therefore, these data demonstrated that immature cartilaginous cells were not susceptible to damage during the frozen storage and did not lose TNF-α-mediated responses under long cultivation in vitro. To facilitate the cell seeding procedure, ready-to use cartilaginous cells were developed. The principle of ready-to use cells is simply to restore and dispense batches of cryopreserved cells directly into assay plates. In order to prepare ready-to-use cells, we identified optimal freezing media that contains 70% of FBS and 5% DMSO. Low DMSO concentration allowed us to skip the DMSO washing step and, thus, directly seed cells in a 96-well assay plate. Comparing cryopreserved cells that were washed out of DMSO before seeding with cells that were directly seeded in 96-well plates revealed insignificant differences in response to TNF-α stimulation (Figure 4b). The time of cell priming by serum deprivation was also optimized. In previous publications, researchers have routinely employed chondrocyte priming with serum deprivation, which was usually performed 24 h before TNF-α treatment [16,17]. Serum deprivation is considered to sensitize cells to cytokine treatment. In our experiments, the MTT viability test demonstrated that 24 h of serum deprivation led to ~25% decrease in viability of the cartilaginous cells (Figure 4c, 10% serum vs. 0% serum). It is interesting that TNF-α treatment of serum-deprived cells did not aggravate cell death (Figure 4c, TNF-α) and the viability of cells was even slightly increased in the presence of etanercept (Figure 4c, T+E). In order to minimize negative effects of serum deprivation, we found the optimal time of serum-free priming for cartilaginous cell-based assay. As shown in Figure 4d, the immature cartilaginous cells primed with serum deprivation for 2 h appear to give slightly higher IL-6 expression compared to 24 h serum-deprived cells. The low level of IL-6 expression in 24 h serum-deprived cells could be associated with their reduced viability as it has been shown by the MTT test (Figure 4c). Thus, the procedure of freezing, resuscitation and priming of primary cartilaginous cells was optimized. The above-mentioned results allowed us to design the optimal TNF-α stimulation scheme which consists of: (a) first day: direct seeding of ready-to-use, cryopreserved cartilaginous cells in a 96-well plate; (b) third day: priming and TNF-α stimulation of cells in the presence of compounds with TNF-α inhibiting activity; (c) fourth day: collection of media and evaluation of IL-6 expression by ELISA.

## 4. Discussion

In the context of applicability of human chondrocytes for in vitro efficacy testing of TNF-α inhibitory drugs, it has been shown that freshly passaged human mature chondrocytes are able to change the gene expression profile in response to TNF-α stimulation [16,17]. The exposure of chondrocytes to TNF-α resulted in remarkable changes of gene expression of cytokines, chemokines, and different matrix-remodeling enzymes such as IL-6, IL-8, IL-32, IL-1B, IL1RA, TNF, MCP1, CCL123, MMP1, MMP3, MMP13, ADAMTS4, etc. The use of TNF-α inhibitors, such as etanercept and infliximab, resulted in the decrease in TNF-α stimulated expression of the above-mentioned genes [16]. The mature chondrocytes used in these studies were isolated from either postmortem cartilage of adult donors or patients scheduled for orthopedic replacement surgery due to a pathology. The effect of both TNF-α and anti-TNF-α inhibitors on the gene expression of human chondrocytes was evaluated by qRT-PCR. The above chondrocyte-based assay for TNF-α inhibitor evaluation has specific limitations that should be taken into account: a limited number of passages due to low proliferative activity of the mature chondrocytes, donor-to donor disease variability, the use of constantly cultivated (not cryopreserved) cells. We succeeded in dealing with these limitations by using unique cartilaginous cells with high proliferative activity, which did not lose their TNF-α-mediated response after both long durations in vitro and through cryopreservation. A high proliferative potency of immature cartilaginous cells isolated from cartilages of supernumerary digits of juvenile donors was demonstrated in recent studies devoted to cartilage tissue engineering [29]. Another advantage of using cartilaginous cells from juvenile donors is that donors had no history of arthritis. Thus, the isolated cartilaginous cells had no history of activation of inflammatory genes, which makes them more suitable for development of a cytokine assessment bioassay than cells obtained from adult donors. Amputated supernumerary digits are considered postsurgical biomaterial waste and at the same time are an attractive cell source to amplify a large number of one-donor cells. Since a gene expression profile of monolayer cultured chondrocytes changes when the number of passages are increased [27,30], we compared the effect of TNF-α stimulation on a monolayer of cultivated immature cartilaginous cells with respect to increasing the passage number. Our results showed that immature cartilaginous cells up to passage 22 do not lose their IL-6 expression response to TNF-α stimulation. The use of a continuous cell culture requires considerable time and technical support for maintenance and monitoring of the cell culture. Banking of in vitro cartilaginous cells makes them comparable to immortal cell lines that are commonly used for cell-based assays. The utilization of ready-to-use, cryopreserved cells for cell-based screening technologies has the potential for substantial cost and time savings [31,32,33]. Extended amplification of cells in vitro followed by cryopreservation allowed us to produce both master and working cell banks that are suitable for screening of a large library of compounds. The ready-to-use vials of cells, thawed and seeded directly into an assay plate, greatly simplified the assay performance. Since the cartilaginous cells isolated from all tested donors, without exception, were showing TNF-α-specific response, large quantities of cells can be also obtained quickly if pooled cells are used from several donors and prepared in advance as homogeneous batches. The assay could be used not only for screening of new compounds with TNF-α inhibitory activity, but also for validation of biological activity of drug batches and comparative studies of multiple biosimilars.

## 5. Conclusions

This work represented a case study describing the development of a screening assay for the evaluation of TNF-α inhibitors using ready-to-use, cryopreserved, human cartilaginous cells. A disease-relevant, convenient and reliable method for the screening of compounds with TNF-α inhibitory activity was developed.

## 6. Patents

Patent RF № 2683277.

## Figures and Tables

**Table 1:** ILl-6 expression by cells from different donors.

Donors	Control 1	TNF-α 2	TNF-α+E 3	*p*_ANOVA_	*p*_1-2_	*p*_1-3_	*p*_2-3_
D1	154.29 ± 4.32	371.70 ± 26.91	158.15 ± 17.14	<0.001	0.001	0.935	<0.001
D2	47.20 ± 31.48	649.21 ± 11.28	70.70 ± 25.06	<0.001	<0.001	0.750	0.012
D3	49.85 ± 23.68	714.87 ± 84.26	85.84 ± 95.79	0.005	0.074	0.929	0.043
D4	15.24 ± 0.11	772.67 ± 94.69	31.70 ± 10.06	<0.001	0.011	0.212	0.011
D5	8.24 ± 0.23	867.10 ± 214.78	18.77 ± 7.14	0.001	0.042	0.251	0.043
D6	14.55 ± 1.20	861.39 ± 124.85	14.78 ± 3.78	<0.001	0.015	0.999	0.015
D7	75.77 ± 9.91	1117.99 ± 9.42	88.33 ± 33.93	<0.001	0.045	0.933	0.020
D8	189.68 ± 32.68	1320.40 ± 139.69	53.09 ± 8.54	0.001	0.077	0.154	0.081
D9	38.10 ± 6.07	1323.68 ± 81.07	57.36 ± 27.39	<0.001	0.003	0.633	0.001
D10	4.11 ± 0.07	1483.77 ± 250.76	14.84 ± 1.86	<0.001	0.020	0.020	0.020

Group-wise comparisons of the donor’s variables. ANOVA was used to determine significance, post-hoc comparisons were performed using Dunnett T3 test. The values represent mean ± SD. D: donors.
